# Diabetes and Thrombosis: A Central Role for Vascular Oxidative Stress

**DOI:** 10.3390/antiox10050706

**Published:** 2021-04-29

**Authors:** Aishwarya R. Vaidya, Nina Wolska, Dina Vara, Reiner K. Mailer, Katrin Schröder, Giordano Pula

**Affiliations:** 1 Institute of Clinical Chemistry and Laboratory Medicine, University Medical Center Hamburg-Eppendorf, D-20246 Hamburg, Germany; aishwarya.vaidya@studium.uni-hamburg.de (A.R.V.); n.wolska@uke.de (N.W.); r.mailer@uke.de (R.K.M.); 2 Biotherapeutics Division, National Institute for Biological Standards and Control (NIBSC), Medicines and Healthcare Products Regulatory Agency (MHRA), London EN6 3QG, UK; Dina.Vara@nibsc.org; 3 Institute of Cardiovascular Physiology, Goethe-University, D-60596 Frankfurt, Germany; Schroeder@vrc.uni-frankfurt.de

**Keywords:** diabetes, oxidative stress, platelet hyperactivity, NADPH oxidase, NFkB, thrombosis, fibrinolysis, endothelial dysfunction

## Abstract

Diabetes mellitus is the fifth most common cause of death worldwide. Due to its chronic nature, diabetes is a debilitating disease for the patient and a relevant cost for the national health system. Type 2 diabetes mellitus is the most common form of diabetes mellitus (90% of cases) and is characteristically multifactorial, with both genetic and environmental causes. Diabetes patients display a significant increase in the risk of developing cardiovascular disease compared to the rest of the population. This is associated with increased blood clotting, which results in circulatory complications and vascular damage. Platelets are circulating cells within the vascular system that contribute to hemostasis. Their increased tendency to activate and form thrombi has been observed in diabetes mellitus patients (i.e., platelet hyperactivity). The oxidative damage of platelets and the function of pro-oxidant enzymes such as the NADPH oxidases appear central to diabetes-dependent platelet hyperactivity. In addition to platelet hyperactivity, endothelial cell damage and alterations of the coagulation response also participate in the vascular damage associated with diabetes. Here, we present an updated interpretation of the molecular mechanisms underlying vascular damage in diabetes, including current therapeutic options for its control.

## 1. Introduction

Diabetes mellitus (DM) is a heterogeneous metabolic disorder characterized by persistent hyperglycemia (HG), which can be diagnosed by quantifying glycated hemoglobin (HbA1c) in peripheral blood. The HbA1c test provides information about glycemic levels in the 3 months preceding blood collection and allows one to distinguish pre-diabetes (HbA1c between 5.7% and 6.5%) from diabetes (HbA1c > 6.5%). Insufficient release of insulin by pancreatic β cells or loss of responsiveness of cells to insulin are the cause of HG in DM. Two main forms of DM are described: type 1 (or T1DM), which is caused by genetic deficiency in insulin release, and type 2 (or T2DM), which is caused by insulin resistance and has mixed genetic and lifestyle determinants. Poor glycemic control in DM patients is accompanied by altered hematological parameters, such as hypercholesterolemia and dyslipidemia, and by a range of serious health complications, including cardiovascular diseases, nerve damage (neuropathy), kidney damage (nephropathy), eye damage (retinopathy), and limb and other peripheral tissue conditions.

Population studies suggest an increase in DM diagnosis from a current count of over 415 million patients globally to over 600 million in 2042 [1]. Six percent of the world mortality rate (i.e., over 3 million deaths annually) is attributed to complications of diabetes. DM patients display a two- to fourfold increase in mortality caused by cardiovascular diseases as compared to the normal population [2]. The risk of developing microvascular complications is particularly increased with HbA1C > 7.9% (>53 mmol/mol) [3]. Disturbed glycemic control increases the propensity for microvascular complications (e.g., retinopathy, neuropathy, and nephropathy) and contributes significantly to the risk factors for macrovascular events, such as age, physical activity, lifestyle, and ethnicity, leading to cardiovascular disease [4].

Longitudinal studies suggest that women with diabetes (35–59 years) have a higher mortality rate due to vascular occlusion than healthy women. However, men with diabetes have increased risk of developing cardiovascular diseases later in life (59–70 years) [5]. Overall, sexual dimorphism in the cardiovascular risk in diabetes is attributable to a combination of diabetes symptoms like dyslipidemia and hormonal control of vascular balance [6].

The vascular damage associated with DM appears to have multiple and simultaneous causes. Platelet hyperactivity, blood hypercoagulability, reduced thrombolysis, and endothelial damage have all been reported and appear to contribute to vascular frailty in DM patients.

## 2. Platelet Hyperactivity in Diabetes

Platelets are anucleated circulating cells derived from megakaryocytes and function to prevent blood loss resulting from injury (i.e., hemostasis). They patrol the vasculature in healthy individuals and are activated in the presence of vascular insults like endothelial denudation, leading to sub-endothelial collagen exposure, or in response to other injury-dependent stimuli such as thrombin and von Willebrand factor (vWF). Platelets also play a pivotal role in thrombotic and inflammatory pathologies [7]. Increased responsiveness of platelets (hyperactivity) has been suggested as a critical driver for cardiovascular complications of diabetes [8,9]. Platelet hyperactivity is suggested by the detection of increased levels of thromboxane B2 in the urine of T2DM patients [10,11]. Thromboxane B2 is a stable degradation product of thromboxane A2, a secondary agonist released by activated platelets.

**Figure 1:**
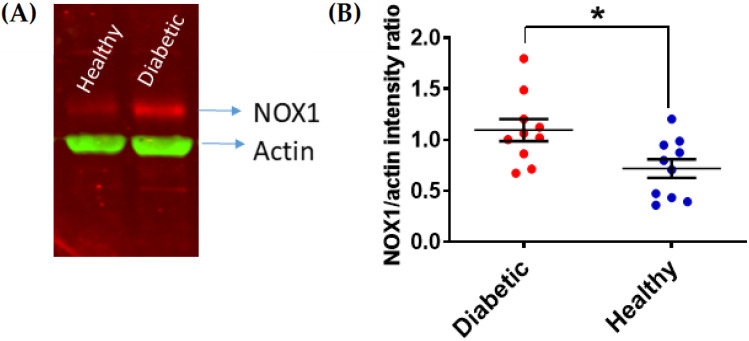
NOX1 upregulation in platelets from DM patients with poor glycemic control. Human platelets from patients with HbA1C > 7.0% were isolated and their levels of NOX1 expression were assessed by immunoblotting as previously described [23]. Actin was co-immunostained as a loading control. NOX1 expression was quantified by densitometry and expression as NOX1/actin ratio (Image J, 47v, Wayne Rasband, National Institute of Health, USA). (**A**) Representative example is shown in (**A**), with the statistical analysis shown in (**B**) (Student’s *t*-test, * *p* < 0.05, *n* = 10).

**Figure 2:**
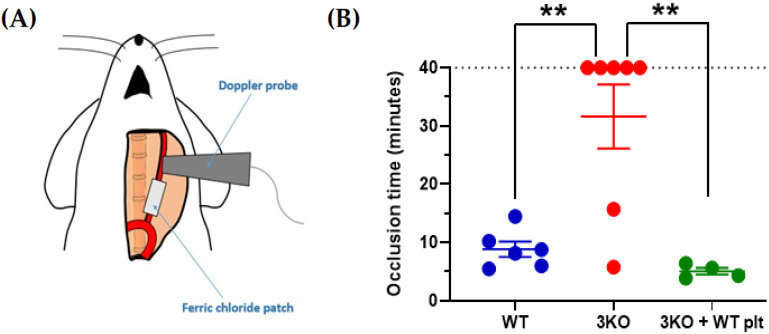
The genetic silencing of NOXs in platelets abolishes thrombotic carotid occlusion induced by ferric chloride. Local application of 5% *w*/*v* ferric chloride induced carotid occlusion (**A**). Doppler ultrasound scanning measured carotid blood flow and complete occlusion times were plotted (**B**). C57BL6/J (WT) were compared to *Nox1^-/-^/Nox2^-/-^/Nox4^-/-^* (3KO) and thrombocytopenic 3KO mice that received infusion of WT platelets. Platelet depletion was induced in 3KO mice by IV injection of the anti-GPIbα antibody R300 (0.2 μg/g body weight). Twelve hours after antibody injection, 6 × 10^8^ platelets from WT mice were IV injected into thrombocytopenic mice (thrombocytopenia was confirmed by blood platelet counting). Data are mean ± SEM and statistical analysis was performed by one-way ANOVA with Bonferroni post-test (** *p* < 0.01, *n* = 4–7).

DM-associated HG affects the expression of key platelet enzymes and receptors at the megakaryocyte stage. The expression levels of the receptor of the negative platelet regulator prostacyclin are decreased in T2DM, which in turn enhances platelet responsiveness [12]. In parallel, P2Y_12_, a key receptor for the secondary agonist adenosine diphosphate (ADP), has been reported to be significantly upregulated in T2DM platelets [13]. The upregulation of P2Y_12_ expression is supported by the activation of oxidative stress-dependent transcription factor nuclear factor-κB (NF-κB) in megakaryocytes. Insulin-like growth factor 1 receptor (IGF1R) is another important receptor upregulated in T2DM patients, which makes platelets from these patients more responsive to IGF1 [14]. Since IGF1 has been described as a positive regulator of platelet signaling and responses [15], the upregulation of IGF1R is likely to contribute to platelet hyperactivity in T2DM. Changes in the membrane expression levels have also been proposed for integrin β3; however, the modulation of the surface expression of this receptor occurs in platelets by microparticle shedding [16]. Work in our laboratory showed that platelets from DM patients with poor glycemic control express significantly higher levels of the pro-oxidant enzyme nicotinamide adenine dinucleotide phosphate (NADPH) oxidase 1 (NOX1) (Figure 1). NOXs have been described as important positive regulators of platelet activity [17,18,19,20]. In view of the pro-thrombotic role of platelet NOXs both in vitro and in vivo (Figure 2), the upregulation of NOX1 in DM patients contributes significantly to platelet hyperresponsiveness. Further studies in this direction are required to elucidate the mechanism of this interaction.

In addition to changes in the proteome of platelets caused by alteration of gene expression, transcription, or protein turnover, DM also regulates platelet function via modulation of different signaling pathways. Markers of platelet activation, such as P-selectin and CD40L, are increased in T1DM and T2DM patients, which suggests raised levels of platelet activation in these patients [21,22]. HG has been shown to directly correlate with the levels of CD40L release (sCD40L) in vitro [22].

High plasma glucose results in increased levels of advanced glycation end products (AGEs) in plasma [24]. AGEs have been shown to activate platelets via activation of the receptor for AGEs (RAGE) [25]. Alternatively, the scavenger receptor CD36 also recognizes AGEs and stimulates platelet activation [26]. Increased pro-coagulant activity of platelets has also been described for T2DM platelets, which was integrin αIIbβ3 dependent [27].

One of the first mechanistic explanations of the hyperactivity of platelets in diabetes suggested a negative regulatory role of insulin in the ADP receptor P2Y_12_ and platelet function. Therefore, insulin resistance and ultimately loss of insulin secretion results in the dysregulation of platelet activation [28]. The insulin-dependent activation of the protein kinase PKB and the modulation of the inhibitory intracellular messenger cAMP support the negative regulatory activity of insulin. Another factor driving platelet hyperactivity can be dyslipidemia, which is often present alongside diabetes. Increased plasma levels of lipids and cholesterol enhance platelet reactivity. Although the evidence was initially only observational [29], recent studies have highlighted the molecular mechanisms linking plasma lipids (low-density lipoprotein, or LDL, in particular) to platelet responsiveness. Typically, dyslipidemia associated with T2DM is accompanied by increased levels of LDL oxidation (ox-LDL) [30]. Ox-LDL has been shown to activate the scavenger receptor CD36 in different cell types, including platelets [31]. The signaling pathway activated by CD36 includes tyrosine kinase- and protein kinase C-dependent activation of NOX2 and generation of reactive oxygen species (ROS), ultimately counteracting the negative regulatory function of the cyclic nucleotides cyclic adenosine monophosphate (cAMP) and cyclic guanosine monophosphate (cGMP). Recent studies from our laboratory highlighted the involvement of both NOX1 and NOX2 in the signaling of ox-LDL [17] and confirmed the negative modulation of the cyclic nucleotide pathways by NOXs [18]. In addition to enzymatic ROS sources, HG causes metabolic overload in platelet mitochondria, which results in the leakage of electrons from the respiration chain and the release of ROS [32]. As a result, protein tyrosine phosphatases are inhibited and the protein kinase signaling pathways are potentiated, which ultimately leads to the potentiation of platelets responses. ROS-dependent inhibition of the protein tyrosine phosphatase Src homology 2 (SH2) domain-containing phosphatase 2 (SHP2) has been shown to lead to increased activity of the protein kinase spleen tyrosine kinase (Syk) and the potentiation of collagen-induced platelet responses [33,34,35].

The role of platelets in vascular health and disease has recently been widened by the discovery of their involvement in the formation of neutrophil extracellular traps (NETs) [36]. NETs have been shown to contribute significantly to thrombotic diseases [37]. Diabetes has been shown to increase NET formation [38,39]. Further studies are required to ascertain whether platelets are a cause for increased NET formation or whether NET formation contributes to DM-dependent thrombosis by inducing platelet activation and vascular occlusion.

## 3. Coagulation and Fibrinolysis

Increased plasma levels for different coagulation factors have been reported for DM patients [40]. Fibrinogen (factor I), pro-thrombin (factor II), pre-kallikrein, factor V, factor VII, factor VIII, factor X, and factor XI have been detected at higher than normal concentration in the plasma of T1DM and T2DM patients [41]. Some coagulation-related proteins are only elevated in T2DM, such as kininogen, factor IX, and factor XIII [42,43]. Interestingly, activated factor XII is downregulated in T1DM, while it is upregulated in T2DM [44]. Tissue factor (TF) is elevated in both T1DM and T2DM [45], although the plasma concentration of TF in response to experimental HG and hyperinsulinemia (HI) is increased in T2DM but not in T1DM. In addition, several anticoagulant proteins have a reduced plasma concentration in both types of diabetes, including anti-thrombin, protein C, and protein S [46]. Overall, the alteration of plasma levels of coagulation factors promotes the hypercoagulative state of DM patients [47]. Oxidative post-translational modification of plasma proteins in T2DM (especially coagulation factor carbonylation) has been proposed as a novel and important factor promoting the procoagulant state of this disease [48].

In parallel, the dissolution of clots (i.e., fibrinolysis) is decreased in DM. Plasminogen activation to form plasmin is the central biochemical reaction of fibrinolysis. Plasmin is a serine protease that acts to dissolve fibrin blood clots. The activation of plasminogen to form plasmin via tissue plasminogen activator (t-PA) and urokinase plasminogen activator (u-PA) promotes fibrinolysis. Increased cross-fibrin cross-linking caused by HG has been suggested to increase clot strength [49] and reduce the fibrinolytic rate in T2DM patients [50]. In addition, HG in T1DM has been suggested to mediate glycation and other post-translational modifications of plasminogen that prevent its activation and limit the formation of plasmin, which ultimately impairs fibrinolysis [51]. Disease duration appears to have a stronger influence on clot strength and fibrinolysis than glycemia control [52]. The implications of this observation remain to be fully understood.

In T2DM, an increased concentration of plasminogen activator inhibitor-1 (PAI-1) reduces fibrinolysis [53,54]. In addition, the concentration of other inhibitors of fibrinolysis, such as thrombin-activatable fibrinolysis inhibitor (TAFI) [55] and α2-macroglobulin [56], are increased in both T1DM and T2DM. In addition, the concentration of α2-antiplasmin is elevated in T2DM [57], while the literature is inconclusive about α2-antiplasmin in T1DM. Taken together, the above studies suggest that reduced fibrinolysis is a likely source of increased thrombotic risk for DM patients. As the consensus is that hypercoagulation is particularly relevant for venous thrombosis, while platelet hyperactivity participates in the onset and progression of arterial thrombosis, the imbalance between coagulation and fibrinolysis may explain the increased risk of both venous and arterial thrombosis in DM patients.

## 4. Endothelial Cell Dysfunction

The endothelium is a cellular monolayer that lines the whole of the vasculature. One of the key physiological roles of the endothelium is the release of antithrombotic signals, such as nitric oxide (NO) and prostacyclin. Endothelial damage induces thrombotic complications by reducing the bioavailability of the abovementioned antithrombotic substances and exposing subendothelial substances that stimulate blood platelets and coagulation (via exposure of platelet activators such as collagen). Vascular inflammation and endothelial cell damage have also been shown to increase the release of vWF [54] and plasminogen activator inhibitor-1 (PAI-1) [58] by endothelial cells. Therefore, as vWF promotes platelet adhesion [59] and PAI-1 inhibits fibrinolysis [60], the overall effect of DM-dependent endothelial cell damage is the increase in platelet adhesion and clot formation. Ultimately, the endothelial cell-dependent modulation of platelets and fibrinolysis participates in the increase in thrombotic risk for DM patients [9]. Raised systemic levels of vWF are associated with vascular comorbidities, making them cardiovascular risk predictors and diagnostic markers in T2D patients [61].

High plasma glucose results in increased levels of advanced glycation end products (AGEs) in plasma [24] and vascular endothelial cells [62]. Endothelial damage also contributes to thickening of the vascular wall, impairs vasodilation, and results in vessel stiffening [63]. The presence of AGEs promotes the activity of the endothelial receptor for advanced glycation end products (RAGE), which contributes to ROS production [64]. Endothelial oxidative stress in DM also derives from Ras-related C3 botulinum toxin substrate 1 (RAC1) and T cell lymphoma invasion and metastasis (TIAM1)-dependent activation of NOX2 [65] and diacylglycerol (DAG)/protein kinase C (PKC)-dependent phosphorylation and stimulation of the NOX activator p47phox [66]. Importantly, endothelial oxidative stress is associated with the formation of superoxide anion, which directly quenches NO by forming peroxynitrite ions and in turn inhibits endothelial nitric oxide synthase (eNOS), a phenomenon that involves the generation of the endogenous intermediate asymmetric dimethylarginine (ADMA), which is an eNOS inhibitor [67].

In addition to the negative regulation of eNOS activity, endothelial oxidative stress also inhibits prostacyclin synthase [68] and activates the pro-inflammatory transcription factor NF-κB [69]. The upregulation of endothelin-1, thrombomodulin, the adhesion molecules ICAM, VCAM, and E-selectin [70], and the increase in the circulating levels of cytokines like IL-1β and TNF-α and chemoattractants like MCP-1 [71] are amongst the most important pro-inflammatory effects of NF-κB activation.

## 5. Therapeutic Intervention

Antiplatelet drugs are commonly prescribed for DM patients. The cyclooxygenase-1 (COX-1) inhibitor acetylsalicylic acid (ASA), the P2Y_12_ receptor antagonists clopidogrel, prasugrel, cangrelor, and ticagrelor, and the GPIIb/IIIa inhibitors abciximab (ReoPro), eptifibatide (Integrilin), and tirofiban (Aggrastat) are the most utilized antiplatelet therapies in clinical practice. The combination of ASA with another antiplatelet drug is known as dual antiplatelet therapy (DAPT), and ADP receptor inhibitors such as clopidogrel, ticagrelor, and prasugrel are usually combined with ASA [72].

Recent studies have highlighted the poor efficacy of ASA in DM patients. The ASCEND trial revealed almost no difference between the number of serious vascular events between the ASA and placebo participants in high-risk DM patients within a 7-year follow-up period [73]. A recent meta-analysis of 12 randomized studies showed that aspirin for primary prevention only reduces major adverse cardiovascular events by 11% [74]. There is evidence for cardiovascular primary prevention in DM patients by DAPT [75]. Nonetheless, a DAPT regime of 75–100 mg ASA/day and 75 mg clopidogrel/day is only advised for secondary prevention in patients with diabetes [76] or for primary prevention in T1DM and T2DM patients with high cardiovascular risk, at age ≥ 50 years and no risk of bleeding [77]. High risk is defined as a family history of at least one condition amongst atherosclerotic cardiovascular disease, hypertension, dyslipidemia, or albuminuria. The increase in bleeding risk for DM patients limits the adoption of DAPT for preventive purposes and highlights the necessity of alternative therapeutic options [76]. Another recent and important study focusing on DAPT concluded that DM patients have less of a positive response to continued pharmacological treatment compared to non-DM patients [78]. More encouraging results are coming from a yet-to-be-completed study exploring the use of DAPT combining ticagrelor and ASA, which significantly reduces major adverse cardiovascular or cerebrovascular events (MACCEs) compared to treatment with ASA alone [79].

Alternatives to ASA may represent an improvement over existing therapeutic options. The thromboxane synthase inhibitor picotamide did not prove more effective in preventing mortality than aspirin, although it reduced unwanted bleeding side effects [80]. The thrombin inhibitor bivalirudin gave some positive results in the ACUITY study with regard to composite ischemia and major bleeding complications [81]. P2Y_12_ receptor antagonists have also been tested on their own in DM patients, highlighting a complex picture in which, depending on comorbidities, clopidogrel, prasugrel, or ticagrelor are the preferred therapeutic options [82]. Overall, cardiovascular prophylaxis in DM remains an unresolved challenge. A better understanding of the molecular mechanisms increasing the cardiovascular risk for DM patients may help to design novel and more efficacious treatments. For example, the participation of oxidative stress in the vascular dysfunction in both T1DM and T2DM may offer some interventional opportunities. NOX inhibitors have been proven to be effective for vascular protection in DM animal models [83,84,85,86,87,88] and clinical studies in humans are ongoing [89]. Dietary antioxidants have also shown some promise to prevent or slow down vascular degradation in DM and protect against cardiovascular complications of this disease. Polyphenols from mushrooms, tea, coffee, and dark chocolate have shown some degree of effectiveness in controlling platelet hyperactivity, mitochondrial stress, and superoxide formation [90] and decreasing the level of plasma oxidized LDL [91]. Food supplements may therefore be designed that help to protect vascular health in DM patients.

In addition to antiplatelet drugs, anticoagulants have been investigated for the treatment of vascular conditions in DM patients. The factor X inhibitor anticoagulant rivaroxaban has been shown to increase the effectiveness of DAPT (ASA + clopidogrel) against MACCEs in acute coronary syndrome patients [92]. As anticoagulants are generally prescribed for atrial fibrillation [93], recent studies have focused on the comparison between traditional vitamin K inhibitor anticoagulants (e.g., warfarin) and novel oral anticoagulants (NOACs, e.g., apixaban, dabigatran, rivaroxaban, and edoxaban). NOACs display better efficacy against thromboembolism events and major adverse limb events than warfarin in DM patients [94]. In addition, NOACs appear to be safer than warfarin as they are associated with a lower risk of major bleeding [95,96]. NOACs should therefore be considered in the diabetic AF population with a high atherosclerotic burden, although a negative interaction with insulin treatment has been noted, which limits their therapeutic efficacy in DM [89].

Although endothelial cell damage is an established pathological mechanism in diabetes, and despite ongoing clinical studies on the use of NADPH oxidase inhibitors [97], there are no effective drugs for endothelial cell protection that have been adopted in this disease.

## 6. Conclusions

**Figure 3:**
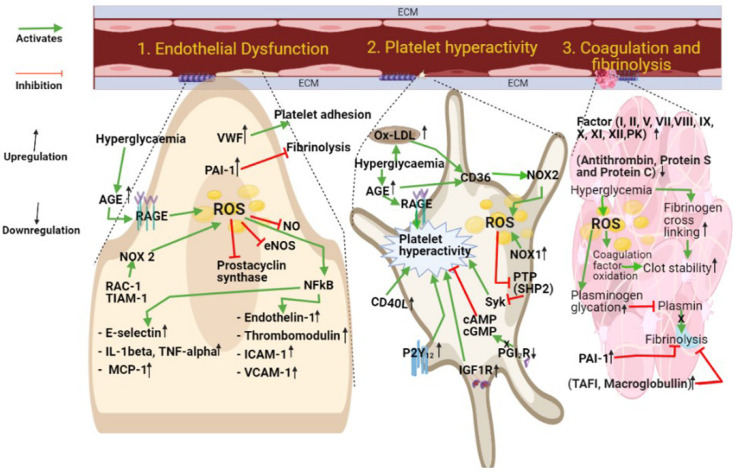
DM-dependent mechanisms underlying vascular damage. Platelet hyperactivity, blood hypercoagulability, and endothelial cell dysfunction are independent drivers of cardiovascular risk in DM patients. Abbreviation list: VWF: Von Willebrand factor; PAI-1: Plasminogen activator inhibitor; NO: Nitric oxide; eNOS: Endothelial nitric oxide synthase; NFκB: Nuclear factor kappa B; Rac-1: Ras-related C3 botulinum toxin substrate 1; TIAM-1: T cell lymphoma invasion and metastasis 1; AGE: Advanced glycation end product; RAGE: Receptor for advanced glycation end products; ROS: Reactive oxygen species; ICAM-1: Intercellular adhesion molecule 1; VCAM-1: Vascular cell adhesion molecule; Ox-LDL: Oxidized low-density lipoprotein; CD36: Cluster of Differentiation 36; NOX1: NADPH oxidase1; NOX2: NADPH oxidase 2; CD40L (Cluster Differentiation 40 Ligand: CD154 (Cluster of Differentiation 154); PTP: Protein tyrosine phosphatase; SHP-2: Src homology region 2 domain-containing phosphatase-2; cAMP: Cyclic adenosine monophosphate; cGMP: Cyclic guanosine monophosphate; PGI2R: Receptor of prostacyclin; IGF-1R: Insulin-like growth factor receptor; PK: Pre-kallikrein; TAFI: Thrombin-activatable fibrinolysis inhibitor.

Taken together, the correlation between vascular frailty and DM has been described, which underlies the increased incidence of cardiovascular diseases in both T1DM and T2DM. Multiple mechanisms have been suggested affecting platelets, the coagulation cascade, or the physiology of the vascular endothelium (Figure 3). Although a significant amount of research in recent years has led to important advances in our understanding of the causes for increased cardiovascular risk in DM, several questions remain unanswered in this area of medical research. In particular, there is an urgent need to improve the therapeutic options available for the clinical management of cardiovascular diseases in DM. An accelerated translation of preclinical discoveries into effective clinical tools via targeted clinical studies appears to be the only viable direction that the biomedical research community has, to resolve this unmet clinical challenge.

## Data Availability

Data in this article will be shared on request to the corresponding author by email.
